# Dietary cauliflower (*Brassica oleracea* var. botrytis) mitigates benzo[a]pyrene-induced oxidative stress, immune dysfunction, and tissue damage in Nile tilapia

**DOI:** 10.1038/s41598-026-57004-4

**Published:** 2026-06-17

**Authors:** Zeinab El-Bouhy, Afaf N. Abdel Rahman, Fatma A. S. Mohamed, Mohamed W. A. Elashhab, Tarek Khamis, Rasha M. Reda

**Affiliations:** 1 Department of Aquatic Animal Medicine, Faculty of Veterinary Medicine, Zagazig University, PO Box 44511, Zagazig, Egypt; 2 Department of Aquatic Health and Diseases, National Institute of Oceanography and Fisheries, NIOF, Cairo, Egypt; 3 Department of Pharmacology, Faculty of Veterinary Medicine, Zagazig University, PO Box 44511, Zagazig, Egypt

**Keywords:** Benzo[a]pyrene, Cauliflower, Feed additives, Genotoxicity, Physiological status, Nile tilapia, Biochemistry, Microbiology, Physiology, Zoology

## Abstract

Benzo[a]pyrene (BaP) is a ubiquitous polycyclic aromatic hydrocarbon that has been reported to induce immunotoxicity and oxidative stress in fish. In the current study, the potential protective efficacy of dietary cauliflower powder (CAF; *Brassica oleracea* var. botrytis) is investigated in Nile tilapia (*Oreochromis niloticus*) subjected to water-borne BaP exposure. Fish (initial body weight 35.45 ± 5.33 g; *n* = 280) were randomly assigned to seven groups (*n* = 40/group): control, acetone (12.25 µL/L), CAF0.5 (0.5%), CAF1 (1%), BaP (12.25 µg/L), CAF0.5 + BaP, and CAF1 + BaP, and maintained on their respective regimens for 30 days. Exposure to BaP induced 30% cumulative mortality, accompanied by distinct clinical-behavioral abnormalities, hepato-renal dysfunction, and depleted total protein fractions. Concurrently, stress biomarkers (glucose and cortisol) and lipid peroxidation (malondialdehyde) were markedly elevated. BaP exposure also suppressed antioxidant-immune responses, caused severe hematological alterations, disrupted splenic gene expression, and provoked pronounced histopathological lesions across multiple tissues (liver, brain, gills, and muscle). This damage correlated with high BaP bioaccumulation in muscle tissue and compromised resistance against *Aeromonas hydrophila* infection. Remarkably, dietary CAF intervention, particularly at the 1% inclusion level, significantly counteracted these toxic effects (*P* < 0.05). CAF supplementation reduced mortality, restored biochemical and immune profiles, lowered muscle BaP residues, and partially preserved normal tissue architecture. Furthermore, CAF-fed fish exhibited significantly enhanced post-challenge survival rates against *A. hydrophila*. Overall, these findings demonstrate that dietary CAF supplementation serves as an effective nutritional strategy to mitigate BaP-induced toxicity and support fish health in contaminated aquaculture environments.

**Supplementary Information:**

The online version contains supplementary material available at 10.1038/s41598-026-57004-4.

## Introduction

*Oreochromis niloticus* (Nile tilapia) is one of the most important aquaculture fish species globally. Its high protein content, strong market demand, and adaptability to diverse environmental conditions, particularly in Africa and Asia, contribute significantly to food security and livelihoods^1–3^. However, the sustainability of this sector faces a serious challenge due to ecosystem exposure to various pollutants, such as industrial effluents, agricultural runoff, and urban waste)^4–6^.

Among the most hazardous environmental pollutants that threaten aquatic ecosystems are polycyclic aromatic hydrocarbons (PAHs), which pose a serious concern to ecosystem stability and public health^7,8^. The U.S. Environmental Protection Agency has classified benzo[a]pyrene (BaP), one of the PAHs, as a priority pollutant due to its well-documented carcinogenic, immunotoxic, and mutagenic effects, even at trace levels^9,10^. BaP enters and accumulates in aquatic environments due to human activities, including industrial effluents from fossil fuel processing and oil refining, as well as the combustion of organic materials^7,11,12^. Due to its lipophilic nature and chemical stability, BaP persists in the environment and bioaccumulates in aquatic organisms, leading to harmful effects on aquatic productivity and fish health^8,13^.

The concentrations of BaP in aquatic systems depend on environmental conditions and pollution sources^14,15^. The selected concentration in this study (12.25 µg/L) was based on monitoring data from Bahr El-Baqar waters that supply fish farms located within the Al-Abbassa area of Egypt. Although this level exceeds typical background levels. It represents a realistic worst-case scenario in highly polluted environments receiving considerable amounts of urban, agricultural, and industrial discharges. Therefore, this concentration is environmentally relevant for assessing ecological risks during severe pollution events rather than average exposure scenarios^16,17^.

BaP induces multifaceted toxicity, primarily through promotion of oxidative stress by increasing reactive oxygen species (ROS) and depleting intracellular glutathione, which leads to severe damage in the liver and various organ cells^18–20^. This oxidative imbalance negatively affects both the innate and adaptive immune responses, weakens defense mechanisms, alters gene expression via the formation of DNA adducts, and activates cytochrome P4501A (CYP1A). Consequently, it leads to genotoxicity, growth abnormalities, metabolic imbalances, persistent inflammation, endocrine abnormalities, and an increased risk of opportunistic infections^21–26^. A promising approach to counteracting these effects is dietary supplementation with bioactive compounds that enhance antioxidant capacity, support immune function, and promote detoxification pathways^27–30^. In this context, cauliflower (*Brassica oleracea*var. botrytis) is a cruciferous vegetable in the *Brassicaceae* family, alongside relatives such as broccoli (*B. oleracea var. italica*) and cabbage (*B. oleracea var. capitata*), known for their rich content of glucosinolates, polyphenols, and vitamins A, C, and E^31–33^.

While many studies have recently investigated cruciferous vegetables in fish, research gaps still exist. Most previous research has focused on broccoli and sulforaphane, leaving limited information regarding the potential of cauliflower (CAF) to mitigate BaP toxicity in *O. niloticus*^34,35^. In addition, previous studies have generally evaluated the effects of dietary exposure^34^ or intraperitoneal injection of BaP^35^; however, the current study employs a chronic BaP waterborne model, which better reflects real-world aquaculture contamination scenarios. Furthermore, while previous studies have often relied on measurements of detoxification enzymes, this study provides a more comprehensive assessment by including oxidative stress markers, immune and hematological responses, gene expression, histopathological alterations, and BaP bioaccumulation.

Thus, this work investigates the protective effects of CAF supplementation on BaP-induced toxicity, specifically by measuring multiple components of physiological performance, immune and antioxidant responses, gene expression, and disease resistance.

## Materials and methods

### Preparation and phytochemical composition of CAF

CAF was sourced from a local market in Egypt and subsequently washed thoroughly to remove dirt, then chopped into pieces (0.6 × 6cm) using a stainless-steel knife. These pieces were soaked in a 2% sodium chloride solution for 10 min to discard the adhering impurities. After that, they were left to dry in the shade for about 7 days, and then finely ground into powder using a mechanical mixer.

For the phytochemical extraction assay^36^, CAF was immersed in ethanol (99.80%) for a 72-h incubation period. A muslin cloth was used to filter CAF after the incubation period, and the filtrate was left exposed in the enclosed space until the ethanol had entirely evaporated. For chromatographic separation, gas chromatography-mass spectrometry (GC–MS; Shimadzu, Japan) was employed with a GC–MS-QP 2010 column. The phytochemical analysis of CAF samples was conducted using a GC–MS oven temperature protocol that started at 40 °C (held for 3 min), increased to 150 °C at 10 °C/min, then ramped again at 5 °C/min to 280 °C, followed by a final hold of 10 min. Helium, a carrier gas, was utilized of 99.999% purity at a 1.0 mL/min flow rate under electronic pressure control, and samples were injected automatically. Mass spectra of unknown compounds were matched with reference spectra in the NIST library (> 62,000 entries). Based on library matching, the identification allowed determination of compound names, structures, and molecular weights.

### Diet formulation and BaP preparation

Three tested diets were created to be iso-nitrogenous and iso-caloric (Table 1). The inclusion levels of CAF were selected based on the previous study of Oke et al.^37^, which investigated *Brassica oleracea* administration in the *O. niloticus* diet at similar levels (0.5–1%) without adverse effects. Finely powdered CAF was incorporated into the basal diet at inclusion levels of 0, 0.5, and 1% (CAF0, CAF0.5, and CAF1, respectively). CAF was thoroughly blended with the other dietary constituents to ensure uniform distribution. The mixture was then mechanically homogenized with the addition of an appropriate amount of water to form a consistent dough. After that, it was pelleted using a laboratory pelletizer into pellets of approximately 1.5 mm. The formed pellets were air-dried (24 h/25 °C) and subsequently stored at 4 °C until use. To fulfill *O. niloticus’* particular dietary requirements, diets were formulated based on NRC^38^ recommendations. The chemical analysis of the formulated diets was performed following previously described methods^39^ as demonstrated in Table 1.

**Table 1:** Ingredients and chemical composition of experimental diets (on a dry basis).

Ingredients (%)	Control diet	CAF0.5	CAF1
Fish meal 66% crude protein (CP)	8	8	8
Ground yellow corn	35	35	35
Soybean meal 48% CP	30	30	30
Corn gluten meal 60% CP	12	12	12
Corn oil	2	2	2
Cod liver oil	2	2	2
Vitamin premix *	1	1	1
Mineral premix **	2	2	2
Wheat bran	8	7.5	7
CAF powder	0	0.5	1
Total	100	100	100
Chemical analysis (%)			
CP	31.07	31.00	31.05
Crude fiber	4.36	4.45	4.35
Crude lipids	7.21	7.20	7.18
Nitrogen-free extract ^#^	51.42	51.30	51.20
Ash	5.94	6.05	6.22
Gross energy (kcal/kg)	4544	4535	4532

CAF: cauliflower plant. * Each kg of vitamin premix contains: vitamin A, 8,000,000 IU; vitamin E, 7 g; vitamin D3, 2,000,000 IU; biotin, 50 mg; pantothenic acid, 7 g; vitamin K3, 1.50 g; folic acid, 0.7 mg; vitamin B12, 7 mg; nicotinic acid, 20 g; vitamin B2, 3.50 mg; vitamin B6, 1 g; vitamin B1, 0.70 g. ** Each kg of mineral premix contains: zinc sulfate, 4 g; sodium selenite, 70 mg; copper sulfate, 2.70 g; manganese sulfate, 5.30 g; cobalt sulfate, 70 mg; iron sulfate, 20 g; calcium iodate, 0.34 g; calcium hydrogen phosphate up to 1 kg. ^#^ Nitrogen-free extract was calculated by difference: 100 – (crude protein + crude fiber + crude lipids + ash).

In addition, BaP was acquired from the El-Shark company (Cairo, Egypt) as a ready-made solution pre-dissolved in acetone (1000 µg BaP/mL). To achieve the target nominal exposure concentration of 12.25 µg/L, a volume of 12.25 µL of stock was added for every liter of aquarium water. Accordingly, the acetone concentration in the water ended up at 12.25 µL/L (0.01225 mL/L). Acetone worked as the carrier solvent so BaP would spread evenly in the water.

### Fish rearing and animal ethics

*O. niloticus* weighing 35.45 ± 5.33 g was obtained from the National Institute of Oceanography and Fishers’ fish farm in Egypt. For 15 days, the fish were housed in well-ventilated 100 L aquaria (80 × 60 × 40 cm; 10 fish per tank) containing dechlorinated water. The fish were thoroughly inspected to verify good health status. Fish received a basal diet three times a day with the daily ration corresponding to 3% of their body weight, adjusted weekly according to tank biomass.

The aquarium’s water was refreshed twice weekly. Measurements of the physicochemical indicators of the raising were performed daily, including temperature (28 ± 2.00 °C), ammonia (0.01 ± 0.005 mg/L), pH (8 ± 0.50), and dissolved oxygen (6 ± 0.50 mg/L). Moreover, the trial methodology was certified by Zagazig University’s Institutional Animal Care and Use Committee under a ZU-IACUC/2/F/125/2023 approval code.

### Experimental design

Fish (*n*=280) were randomly assigned to seven experimental groups, comprising four replicate tanks per group (10 fish per tank), including control, acetone, CAF0.5, CAF1, BaP, CAF0.5+BaP, and CAF1+BaP for 30 days. The control and acetone solvent groups were fed on the CAF0 diet and were exposed to acetone at 0 and 12.25 µL/L levels, respectively. The CAF0.5 and CAF1 treatments were provided with CAF0.5 and CAF1 diets, respectively, and were not subjected to BaP. The BaP, CAF0.5+BaP, and CAF1+BAP treatments received CAF0, CAF0.5, and CAF1 diets, respectively, and were exposed to 12.25 µg/L of BaP (delivered by adding 12.25 µL of stock solution per liter of water). The acetone concentration (12.25 µL/L) was justified as non-toxic, being significantly below the OECD^40^ recommended limit of 100 µL/L for aquatic toxicity assays.

Three times (9:00, 13:00, and 17:00 h) daily, the fish were fed the tested diets at a rate of 3% of body weight per day. During each feeding, fish were fed gradually until they appeared satisfied. If they didn’t finish their meal, we made sure to offer the leftover portion in the next feeding session that day. Feed amounts were modified weekly according to average tank weight to ensure uniform feeding across treatments. A semi-static exposure system was used for BaP administration. The entire tank’s water (exposure medium) was completely renewed twice weekly using dechlorinated water, and freshly prepared BaP solution was added after each renewal to maintain relatively stable exposure concentrations and minimize losses due to degradation and adsorption. Though the concentration of BaP was not analytically confirmed, this protocol aligns with semi-static practice in aquatic toxicology exposure designs^41,42^. Furthermore, this nominal concentration (12.25 µg/L) was intentionally chosen to simulate a worst-case environmental pollution scenario, thereby providing a stringent challenge for assessing the maximum protective potential of dietary CAF under severe toxicological stress. Over a period of 30 days, fish mortality and clinical observations were monitored.

### Sampling

Following 30 days, the fish were fasted for 24 h, and the bleeding started. Benzocaine solution (100 mg/L) was used to sedate fish^43^. From each replicate tank (*n* = 4 tanks per group), three fish were randomly selected (totaling 12 fish per treatment group) for biological sampling. The tank was designated as the experimental unit for statistical analysis, and individual fish were regarded as sub-samples within each tank to calculate tank means. After that, caudal blood vessels were punctured, and samples were divided into two subsets. Anticoagulant-devoid syringes were used to recover the first set, and blood was left to coagulate (4 °C). Serum was then extracted for biochemical, oxidant/antioxidant, and immuno-analyses by centrifuging for 10 min at 1198 × g. Meanwhile, heparinized syringes were used to drain the second batch for hematological investigations. Additionally, the fish were euthanized by being exposed to a solution of 300 mg/L benzocaine^44^ to gather their tissues. Specimens (3 fish per tank; 12 fish per treatment group) of spleen, brain, and muscles were removed for gene expression, stress/neuro, and BaP residue diagnostics, respectively. Additionally, for histological analysis, the liver, brain, gills, and muscles were collected in 10% buffered formalin (3 fish per treatment, sampled randomly from the experimental replicates). All assays were carried out in triplicate following the manufacturer’s recommendations. Standard calibration curves were employed to ensure consistency and accuracy of the data.

### Biochemical assays

For determination of serum creatinine, urea, alanine aminotransferase (ALT), alkaline phosphatase (ALP), and aspartate aminotransferase (AST) concentrations, the Bio-diagnostic Co diagnostic kits (Cat. Nos. CR 1250, UR 2110, AL 1031, AP 1020, and AS 1061), respectively, were utilized.

The creatinine concentration was measured^45^ by mixing 50 μL of serum with 1 mL of chromogen reagent, followed by the addition of 10 μL of creatinine amidohydrolase (8.84 mmol/L). After incubating for 30 min at ambient temperature, the sample absorbance was recorded at 510 nm. To estimate urea concentrations, the procedure of Chaney et al.^46^ was used. The method depends on urease activity, where urea is hydrolyzed to produce ammonia and carbon dioxide. The ammonia then reacts with designated reagents to yield a colored complex, with its intensity corresponding to the urea level in the sample. Spectrophotometric measurement of the chromogen was performed at 578 nm.

ALT and AST activities were measured^47^ using 50 μL of serum incubated with 0.25 mL of the respective substrate buffer (L-alanine for ALT, 30 min; L-aspartate for AST, 60 min) at 37 °C. After incubation, 0.25 mL of 2,4-dinitrophenylhydrazine was added, and after 20 min at room temperature, about 2.5 mL of 0.4 N sodium hydroxide was administered. The mixtures were thoroughly mixed, incubated for 10 min, and the optical density was measured at 505 nm. Moreover, ALP activity was analyzed by applying the methodology of Bowers et al.^48^. This method depends on the hydrolysis of p-nitrophenyl phosphate to p-nitrophenol that reacts with the kit reagents to produce a colored complex. Color intensity, measured at 405 nm, is proportional to the ALP activity.

Protein profiling comprising albumin and total protein was determined spectrophotometrically by cellulose acetate electrophoresis (Helena Laboratories, USA), in line with a previous method^49^. In brief, serum samples were loaded onto cellulose acetate strips pre-treated with buffer, and electrophoresis was performed under standard conditions. Following separation, the strips were stained to visualize the protein fractions, and the band densities were measured spectrophotometrically to quantify albumin and total protein concentrations. Globulin levels were estimated by deducting albumin from total protein^50^.

Spectrophotometrically, the glucose level (Cat No.: GL 1320) was quantified by commercial kits of Bio-Diagnostics CO., following the Trinder^51^ approach. In this assay, glucose is oxidized to gluconic acid through glucose oxidase, forming hydrogen peroxide (H_2_O_2_), which reacts with phenol-aminophenazone, generating a pink color in the presence of peroxidase. Glucose levels were determined based on the intensity of the developed color. To assess cortisol levels, a commercial ELISA kit (Cat No.: MBS704055; My-BioSource kits, San Diego, USA) was used based on a previous methodology^52^. Briefly, duplicate samples containing cortisol antibodies were added to antigen-coated wells and incubated (37 °C/40 min). After washing, horseradish peroxidase conjugate was introduced and incubated (30 min/37 °C). Following a second wash, tetramethylbenzidine substrate was added and incubated for 20 min at 37 °C. A stop solution was then applied, and the optical density was measured at 450 nm to determine cortisol concentrations.

### Oxidant/antioxidant and immune assays

The malondialdehyde (MDA), catalase (CAT), glutathione S-transferase (GST), and superoxide dismutase (SOD) were assessed in the serum utilizing the diagnostic kits from Bio-diagnostics CO., with Cat. Nos. MD 2529, CA 2517, GT 2519, and SD 2521, respectively. For this, MDA concentration at 534 nm was assessed using the thiobarbituric acid (TBA) assay as reported by Ohkawa et al.^53^. In this analysis, MDA reacts with TBA in acidic conditions to form a pink chromogen at 95 °C for 30 min. CAT activity was evaluated using a prior technique^54^. Briefly, potassium phosphate buffer (0.5 mL; pH 7.0) was mixed with 0.1 mL of H_2_O_2_, followed by the addition of 0.2 mL of chromogen and 0.5 mL of the peroxidase–4-aminoantipyrine. After incubation (1 min/25 °C), the decomposition of H_2_O_2_ was evaluated at 510 nm.

In addition, the GST activity was determined by Habig et al.^55^ on the basis of the conjugation of reduced glutathione with 1-chloro-2,4-dinitrobenzene (CDNB), forming a thioether product. The reaction mixture generally consisted of CDNB, potassium phosphate buffer (pH 7.4), enzyme extract, and reduced glutathione. The test was started by adding CDNB, and the increase in absorbance was monitored continuously over time at 340 nm to calculate GST levels. Following the protocol of Nishikimi et al.^56^, SOD was determined based on the extent of inhibition to phenazine methosulfate–driven reduction of nitroblue tetrazolium. The homogenization buffer for SOD consisted of 100 mM potassium phosphate (pH 7.0) supplemented with 2 mM EDTA. The reaction was incubated at 25 °C for 5 min, and the absorbance was assessed at 560 nm.

The calorimetric evaluation of serum antiprotease activity was carried out using the Bowden et al.^57^ procedure. In this regard, 10 μL of serum was incubated with 20 μL of 0.1% trypsin for 5 min, followed by the addition of 500 μL of BAPNA substrate and 470 μL of buffer. Antiprotease activity was represented as trypsin inhibition. Adopting Ellis^58^ technique, a method focused on the lysis of freeze-dried *Micrococcus lysodeikticus* (Sigma-Aldrich Chemie, Germany) was used to estimate the serum lysozyme activity level. Serum (0.2 mL) was mixed with 0.25 mg/mL bacterial suspension (pH 6.2) and incubated at 25 °C for 5 min. The optical density at 450 nm was recorded every minute for 5 min. Lysozyme concentration was quantified using a standard curve prepared with serial dilutions of lyophilized chicken egg-white lysozyme (Sigma, USA).

Furthermore, a prior approach^59^ using the Griess reaction was implemented to analyze serum nitric oxide (NO) spectrophotometrically. In short, serum samples (100 µL) were treated with Griess reagent, followed by incubation for 10 min at 27 °C. At an absorbance of 546 nm, the NO levels were calculated. The level of immunoglobulin M (IgM) was assessed using a commercial ELISA kit (My-BioSource kits; Cat No. MBS042385) according to the kit guidelines with a detection range of 2.50–80 mg/dL (equivalent to 25–800 µg/mL). Standards and serum samples were applied to antigen-coated 96-well plates, incubated with enzyme conjugates, and washed. The substrate reaction was measured spectrophotometrically at 450 nm, and IgM concentrations were estimated.

### Hematological assays

An automatic hematology analyzer specifically designed for veterinary use (Hospitex Diagnostics, Italy; model VET-Hemato, calibrated for fish blood) was applied to analyze the hematological parameters. The analyzer provides automated differential counts, calculated indices, and ensures high precision for non-mammalian species. The following hematological variables were assessed: total white blood cell counts (WBCs), differential WBC counts, red blood cell counts (RBCs), hemoglobin (Hb), packed cell volume (PCV), mean corpuscular hemoglobin concentration (MCHC), and mean corpuscular volume (MCV).

### Gene expression assay

As per the manufacturer’s guidelines, 1 mL of QIAzol l (Qiagen, Germany) was employed to extract total RNA from splenic tissue specimens (30 mg). The extracted RNA was subjected to DNase I treatment to eliminate residual genomic DNA, and subsequently incubated with the addition of an RNase inhibitor to ensure the integrity of RNA. For complete cellular disruption, specimens were homogenized in QIAzol reagent (1 mL) through employing a tissue homogenizer. After homogenization, chloroform was added (200 µL), and the mixture was vortexed for 30 seconds, followed by centrifugation (12,000 rpm/15 min). The aqueous phase was carefully recovered and mixed with isopropanol (400 µL; Sigma-Aldrich, USA) for RNA precipitation, followed by centrifugation for 10 min. The precipitated RNA pellet was washed with 75% ethanol, air-dried, and finally reconstituted in RNase-free water. At specific absorbance (260/280 and 260/230 nm), both RNA concentration and purity were determined using a spectrophotometer (Quawell UV–Vis Q3000). RNA integrity was estimated by agarose gel electrophoresis, and 260/280 ratios of 1.8–2.0 and 260/230 ratios were acceptable for subsequent analyses.

The complementary DNA was created via an Applied Biosystems reverse transcriptase kit (California, USA), and the total RNA (1000 ng) was reverse transcribed in line with the manufacturer’s standards. The expression of heme oxygenase-1 (*ho-1*), nuclear factor kappa B p65 (*nf-κb-p65*), C/EBP homologous protein (*chop*), and c-Jun N-terminal kinase (*jnk*) was evaluated using the Sangon Biotech (Beijing, China) primers provided in Table 2. To evaluate primer efficiency, standard curves were derived from serial cDNA dilutions, resulting in efficiencies of 93–99% with R^2^ ≥ 0.98 for each gene. Melting curve analysis was performed to exclude the presence of primer dimers and non-specific products. The Rotor-Gene Q2 Plex real-time thermal cycler from Qiagen (Germany) was used for quantitative real-time PCR (RT-qPCR). Based on a prior study^60,61^, the cycling conditions were assessed. Elongation factor 1-alpha (*ef-1α*) served as a reference gene based on its stable expression and limited variation across groups, as determined by (https://genorm.cmgg.be/). The expression data were evaluated using the 2^−ΔΔCT^ approach^62^ by determining relative fold changes with respect to the reference gene.

**Table 2:** Gene primers for quantitative real-time PCR amplification.

Target gene	Primer sequence	Melting temperature (°C)	Size (bp)	Primer efficiency (%)	Accession number
*ho-1*	F: ACAGGTTCTTGGTCGGATCG R: CACACTGTTCATTCGGCTGC	59.75 60.11	133	98.50	XM_013272963.3
*nf-κb-p65*	F: GAGAAAGCGGACAGGAGACAT R: AGCAGCTTGTGGAGGCTTG	59.79 60.60	133	93.68	XM_025905614.1
*chop*	F: GACACAGGAGGGGCAAAACT R: GCTCCTTCTGGAAGCACAAA	60.18 58.39	81	98.05	XM_013275519.3
*jnk*	F: AAAGCGTGGTGGAGTCTCTG R: CTCCCTCTCAGCCTCTTCCT	60.03 59.97	104	97.82	XM_005455390.4
*ef-1α*	F: CTACGTGACCATCATTGATGCC R: AACACCAGCAGCAACGATCA	59.45 60.53	106	97.81	XM_019350634.2

*ho-1*, heme oxygenase-1; *nf-κb-p65*, nuclear factor kappa B p65; *chop*, C/EBP homologous protein; *jnk*, c-Jun N-terminal kinase; *ef-1α*, elongation factor 1-alpha; F, forward primer; R, reverse primer.

### Stress-neuro assays

In brain tissues from whole groups, the concentrations of 8-hydroxy-2’-deoxyguanosine (8-OHdG) and acetylcholinesterase (AchE) were measured. The tissues were centrifuged (5 °C, 3000 × g, 15 min) after homogenization in 150 mM NaCl. Following Setyaningsih et al.^63^ approach, the amount of 8-OHdG was assessed using a My-Biosource kit (Cat. No. MBS1601729) at 450 nm. The final 8-OHdG tissue concentrations were expressed as ng/g tissue. Furthermore, using a commercial kit (Cat. No. MBS035436) from My-Biosource and a 450 nm wavelength, the activity of AchE was calculated as per a previous technique^64^. The final AchE enzyme activity was estimated relative to the brain weight and expressed as µmol/g tissue.

### Evaluation of BaP residues

A previous approach of Mastovska et al.^65^, with some modifications, was conducted to determine the BaP residues in fish muscles. Representative specimens (12 per group) were collected from muscles at the close of the 30-day experiment. Samples were thawed and homogenized mechanically. The homogenized tissue (5 g) was mixed with 10 mL of acetonitrile, 1 g of sodium chloride, and 4 g of magnesium sulfate. After 2 min of vortexing, the mixture was spun (5 min/4000 rpm), and the supernatant (acetonitrile layer) was cleaned using dispersive solid-phase extraction with PSA and C18 sorbents to eliminate lipids and co-extracted impurities. The purified extract was evaporated at 40°C under a nitrogen stream and reconstituted in 1 mL dichloromethane, then filtered through a 0.22 PTFE membrane before analysis.

GC–MS (Agilent 7890 GC coupled to a 5977 MSD) was used to quantitatively analyze BaP. On a fused silica capillary column (30 m × 0.25 mm i.d., 0.25 µm film thickness), separation was accomplished. The temperature program for the GC–MS oven was configured to begin at 70°C for 1 min, ramp to 150°C at 20°C/min, then at 10°C/min to 280°C, held for 10 min. As a carrier gas, helium was employed at a 1 mL/min. For the target PAH ions, the spectrometer was run in the selected ion monitoring approach.

Calibration curves were prepared using BaP standards (0.006–1 µg/mL). Procedural blanks, spiked samples, and duplicates were analyzed alongside to ensure method reliability. The method’s limit of detection (LOD) and limit of quantification (LOQ) were 0.002 and 0.006 µg/mL, respectively, based on signal-to-noise ratios of 3:1 (LOD) and 10:1 (LOQ). Accuracy was evaluated through recovery studies at three spiking levels (5%, 100%, and 200% of the target concentration), yielding recoveries of 70–120% with a relative standard deviation (RSD) < 10%, confirming accuracy and precision.

### Histopathological inspection

Tissue fragments were taken from the liver, brain, gills, and muscles after being treated with 10% buffered neutral formalin. Standard histological processes were used to prepare these specimens^66^. To do this, the tissues were first fixed in neutral buffer formalin (10%/48 h), then dehydrated in increasing ethyl alcohol grades (70–100%), and last cleaned in xylene. Melted paraffin wax was then used for tissue fixation, and paraffin Sects. (5 µm) were produced using a Leica® microtome. Hematoxylin and eosin (H&E) stains were subsequently applied for microscopic examination using an AmScope microscope (USA). Liver, brain, and muscle sections were examined at 40 × magnification, whereas gill sections were observed and photographed at 10 × .

### Challenge assay

At the Department of Aquatic Animal Medicine of the Faculty of Veterinary Medicine, Zagazig University, an isolate of *Aeromonas hydrophila* from diseased *O. niloticus* was previously obtained, and its pathogenicity to this species had been established. The automated VITEK 2-C15 system from BioMérieux (Marcy-l’Étoile, France) was deployed to identify this strain at the National Research Centre (Dokki, Egypt). In the current investigation, the same bacterial strain used in the Hassanin et al.^67^ study was employed; the bacterium comes from the same laboratory source, it has been consistently grown and kept under standard conditions, and it is stored in glycerol stocks at − 80 °C.

According to an earlier investigation^67^, in which the lethal dose 50 of *A. hydrophila* was experimentally determined in *O. niloticus* to be 3 × 10^6^ CFU/mL using Finney probit analysis, a sub-lethal inoculum of 1.5 × 10^6^ CFU/mL was applied for the challenge. This dose was selected to ensure a reproducible infection while minimizing the mortality rate. This enables reliable evaluation of the immune system and resistance to the disease using the present experimental method. The dose used in this experiment is quite comparable with what is used in the cited reference in terms of host, infection method, and rearing conditions. The previously proved protocol was followed to prepare the bacterial inoculum. The bacterial concentration was triple checked by using standard plate counts to ensure the reproducibility of the challenge dose. After a 30-day trial, 12 fish from each experimental group were selected for bacterial challenge assay. Fish were intraperitoneally (IP) injected with 0.1 mL *A. hydrophila* suspension (1.5 × 10^6^ CFU/mL). The remaining fish in each group served as controls and received 0.1 mL of sterile phosphate-buffered saline via IP injection. Before the challenge, fish were fasted for 24 h and anesthetized by immersion in benzocaine (100 mg/L). Assigned experimental diets were reinstated 12 h following the inoculation. For 15 days, fish were tracked twice daily to record any mortality or unusual clinical symptoms. The results of mortality and clinical signs noted in the PBS-treated group further confirmed the establishment of an infection model under the present experimental conditions.

### Statistical analyses

Data were analyzed at the tank level, with each tank serving as the experimental unit (*n* = 4 tanks per group) and individual fish within the tanks treated as subsamples to calculate tank means. The assumptions of normality and homogeneity of variance were verified using the Shapiro–Wilk test and Levene’s test, respectively, before parametric testing. To evaluate the main effects of CAF supplementation and BaP exposure, as well as their interaction term (CAF supplementation × BaP exposure), a two-way analysis of variance (ANOVA) was performed. Fixed factors in the model included CAF supplementation and BaP exposure. Tukey’s HSD post hoc range test was applied to investigate the statistically significant differences among treatment means (*P* < 0.05). These data are expressed as means ± pooled standard error (PSE). Additionally, an independent samples t-test was conducted to specifically compare differences between the control and acetone vehicle control treatments; these data are expressed as means ± standard error (SE). Mortality was monitored daily throughout the initial exposure phase and presented as a cumulative percentage for each treatment group. For the subsequent bacterial challenge phase, Kaplan–Meier statistics were applied to estimate treatment-specific survival probability along with a log-rank (Mantel-Cox) test to detect and compare statistical differences among survival profiles of the treatments (*P* < 0.05). All statistical analyses were executed using IBM SPSS Statistics software (version 22; IBM Corp., Armonk, NY, USA).

## Results

### Chemical constituents of the CAF by GC–MS analysis

GC–MS analysis (Table 3) revealed that CAF is predominantly composed of isothiocyanates, particularly phenethyl (19.20%), 3-butenyl (15.90%), 4-pentenyl (13.50%), and benzyl isothiocyanates (11.20%), along with linoleic acid and several terpenes. Such molecules were classified into major groups, and their presence may partly explain the observed antioxidant and immunological responses.

**Table 3:** Findings of GC–MS analysis of CAF.

Peak	Compounds	Class*	Area (%)	MW	R_t_ (min)	CF
1	Hexanal	Aldehyde	1.82	100	4.52	C_6_H_12_O
2	(Z)-3-hexen-1-ol	Alcohol	3.25	100	8.19	C_6_H_12_O
3	α-pinene	Terpene	0.97	136	10.55	C_10_H_16_
4	Myrcene	Terpene	1.16	136	12.86	C_10_H_16_
5	Limonene	Terpene	2.77	154	15.71	C_10_H_16_
6	Eucalyptol (1,8-cineole)	Terpenoid	0.79	154	16.03	C_10_H_18_O
7	Linoleic acid	Fatty acid	5.65	280	16.80	C_18_H_32_O_2_
8	Stearic acid	Fatty acid	2.10	284	17.45	C_18_H_36_O_2_
9	Linalool	Terpenoid	1.45	154	24.66	C_10_H_18_O
10	3-butenyl isothiocyanate	Isothiocyanate	15.90	129	26.85	C_5_H_7_NS
11	4-methylthiobutyl isothiocyanate	Isothiocyanate	8.91	177	32.10	C_6_H_11_NS_2_
12	3-methylindole	Indole derivative	4.35	131	35.23	C_9_H_9_N
13	4-pentenyl isothiocyanate	Isothiocyanate	13.50	143	36.55	C_6_H_9_NS
14	Caryophyllene	Terpene	3.05	204	39.11	C_15_H_24_
15	Phenethyl isothiocyanate	Isothiocyanate	19.20	163	41.80	C_9_H_9_NS
16	α-farnesene	Terpene	2.98	204	47.25	C_15_H_24_
17	Benzyl isothiocyanate	Isothiocyanate	11.20	149	49.50	C_8_H_7_NS

MW, molecular weight; R_t_, retention time; CF, chemical formula.

*Compounds were classified into isothiocyanates, fatty acids, terpenes/terpenoids, and other volatile compounds based on their chemical nature.

### Mortality and clinical observations

Fish in the control, acetone, and CAF-fed groups showed no mortality or behavioral abnormalities. However, exposure to BaP resulted in a significant increase in mortality (30%) and severe clinical signs, including anorexia, abnormal swimming, fin rot, respiratory manifestations, and pale gills. Supplementation with CAF (0.5 and 1%) in the diets lowered BaP-related mortality (10 and 5%, respectively) and improved the associated clinical symptoms, supporting its protective potential.

### Biochemical responses

Table 4 discloses that the interaction between CAF supplementation and BaP exposure considerably (*P* < 0.05) affected renal-hepatic and stress indices. Dietary CAF alone significantly (*P* < 0.05) enhanced albumin, total protein, and globulin levels and decreased creatinine, urea, ALT, and stress marker without affecting ALP and AST. BaP exposure disrupted these parameters (*P* < 0.001), causing elevations in renal-hepatic and stress indices, alongside reductions in protein profile parameters. CAF supplementation in BaP-exposed fish restored these biochemical indices toward normal, with CAF1 showing the greatest improvement (*P* < 0.001), highlighting dose-dependent hepatoprotective and stress-alleviating effects. In addition, the addition of acetone did not change these variables relative to the control group (supplementary Table 1).

**Table 4:** Biochemical biomarkers of *O. niloticus* fed diets supplemented with CAF and/or exposed to BaP for 30 days.

Parameters	Creatinine (mg/dL)	Urea (mg/dL)	ALT (U/L)	ALP (U/L)	AST (U/L)	Albumin (g/dL)	Total protein (g/dL)	Globulin (g/dL)	Glucose (mg/dL)	Cortisol (ng/mL)
*Effect of CAF supplementation*										
Non-supplemented	1.02^a^	17.02^a^	29.50^a^	44.50	23.00	1.92^b^	2.73^b^	0.81^b^	82.67^a^	64.50^a^
CAF0.5	0.96^ab^	15.94^ab^	26.50^ab^	42.83	21.83	2.27^a^	3.33^a^	1.06^a^	75.50^b^	55.67^b^
CAF1	0.92^b^	15.24^b^	24.17^b^	40.33	19.83	2.29^a^	3.47^a^	1.18^a^	70.50^b^	52.00^b^
*Effect of BaP exposure*										
Non-exposed	0.81^b^	12.99^b^	21.56^b^	37.33^b^	18.56^b^	2.38^a^	3.60^a^	1.22^a^	62.56^b^	45.78^b^
BaP	1.12^a^	19.14^a^	31.89^a^	47.78^a^	24.56^a^	1.94^b^	2.75^b^	0.81^b^	89.89^a^	69.00^a^
*Interaction effect (CAF supplementation x BaP exposure)*										
Control	0.75^c^	12.12^c^	21.00^d^	35.00^c^	18.00	2.23	3.25	1.02	60.00^d^	48.00^d^
CAF0.5	0.84^c^	13.47^c^	22.33^d^	38.33^c^	19.66	2.46	3.76	1.30	65.66^d^	45.33^d^
CAF1	0.83^c^	13.39^c^	21.33^d^	38.66^c^	18.00	2.44	3.79	1.35	62.00^d^	44.00^d^
BaP	1.28^a^	21.91^a^	38.00^a^	54.00^a^	28.00	1.60	2.20	0.60	105.33^a^	81.00^a^
CAF0.5 + BaP	1.08^b^	18.40^b^	30.66^b^	47.33^b^	24.00	2.07	2.90	0.83	85.33^b^	66.00^b^
CAF1 + BaP	1.00^b^	17.09^b^	27.00^c^	42.00^b^	21.66	2.14	3.14	1.00	79.00^c^	60.00^c^
PSE	0.01	0.18	0.53	0.78	0.52	0.05	0.05	0.03	1.07	0.89
*Two-way ANOVA*	*P-values*									
CAF supplementation	0.035	0.006	0.005	0.129	0.081	0.01	< 0.001	< 0.001	0.002	< 0.001
BaP exposure	< 0.001	< 0.001	< 0.001	< 0.001	< 0.001	< 0.001	< 0.001	< 0.001	< 0.001	< 0.001
Interaction	< 0.001	< 0.001	0.002	0.005	0.067	0.350	0.320	0.675	< 0.001	0.006

Data (mean) represent tank-level replication (*n* = 4 tanks per treatment group, with 3 fish subsampled per tank to calculate individual tank means). Values carrying various superscripts within the same column are significantly different (two-way ANOVA, Tukey’s HSD test, *P* < 0.05). Overall pooled standard error (PSE) and *P*-values for main effects (CAF supplementation, BaP exposure) and their interaction are presented.

Groups: control, CAF0.5 (0.5% CAF), CAF1 (1% CAF), BaP (12.25 µg/L), CAF0.5 + BaP, and CAF1 + BaP.

### Oxidant/antioxidant and immune indices

CAF diets increased antioxidant (CAT, GST, and SOD) and immune (antiprotease, lysozyme, NO, and IgM) activities in a dose-dependent manner, and decreased MDA level (Table 5). BaP exposure induced oxidative stress (elevated MDA) and suppressed antioxidant and immune responses (*P* < 0.001). Co-administration of CAF mitigated oxidative damage and enhanced immune function, with CAF1 providing superior protection. In addition, the interaction markedly altered all these variables (*P* < 0.05) except the antiprotease, lysozyme, and IgM levels. Moreover, these parameters were not significantly affected by the addition of the acetone (supplementary Table 2).

**Table 5:** Antioxidant-immune responses of *O. niloticus* fed diets supplemented with CAF and/or exposed to BaP for 30 days.

Parameters	MDA (nmol/mL)	CAT (U/mL)	GST (U/mL)	SOD (U/mL)	Antiprotease activity (U/mL)	Lysozyme (U/mL)	NO (µmol/L)	IgM (mg/dL)
*Effect of CAF supplementation*								
Non-supplemented	14.97^a^	114.17^c^	4.46^c^	4.19^c^	0.36^c^	17.67^b^	122.50^c^	17.00^c^
CAF0.5	12.70^b^	141.67^b^	6.30^b^	8.15^b^	0.58^b^	22.50^a^	144.50^b^	24.17^b^
CAF1	12.10^b^	176.00^a^	7.62^a^	10.25^a^	0.70^a^	24.67^a^	155.33^a^	27.83^a^
*Effect of BaP exposure*								
Non-exposed	12.05^b^	177.44^a^	8.28^a^	11.13^a^	0.73^a^	26.00^a^	170.00^a^	28.67^a^
BaP	14.47^a^	110.44^b^	3.98^b^	3.93^b^	0.36^b^	17.22^b^	111.56^b^	17.33^b^
*Interaction effect (CAF supplementation x BaP exposure)*								
Control	12.23^c^	158.33^c^	7.06^b^	5.85^c^	0.51	23.66	157.66^b^	21.66
CAF0.5	11.80^c^	181.33^b^	8.56^a^	12.46^b^	0.77	26.65	174.00^a^	29.66
CAF1	12.10^c^	192.66^a^	9.20^a^	15.06^a^	0.91	27.67	178.33^a^	34.65
BaP	17.70^a^	70.00^e^	1.86^d^	2.53^e^	0.20	11.66	87.33^d^	12.33
CAF0.5 + BaP	13.60^b^	102.00^d^	4.03^c^	3.83^d^	0.39	18.33	115.00^c^	18.67
CAF1 + BaP	12.10^c^	159.33^c^	6.03^b^	5.43^c^	0.48	21.67	132.33^bc^	21.00
PSE	0.11	1.02	0.09	0.08	0.01	0.58	1.04	0.61
*Two-way ANOVA*	*P-values*							
CAF supplementation	< 0.001	< 0.001	< 0.001	< 0.001	< 0.001	0.001	< 0.001	< 0.001
BaP exposure	< 0.001	< 0.001	< 0.001	< 0.001	< 0.001	< 0.001	< 0.001	< 0.001
Interaction	< 0.001	< 0.001	0.001	< 0.001	0.185	0.151	0.002	0.369

Data (mean) represent tank-level replication (*n* = 4 tanks per treatment group, with 3 fish subsampled per tank to calculate individual tank means). Values carrying various superscripts within the same column are significantly different (two-way ANOVA, Tukey’s HSD test, *P* < 0.05). Overall pooled standard error (PSE) and *P*-values for main effects (CAF supplementation, BaP exposure) and their interaction are presented

Groups: control, CAF0.5 (0.5% CAF), CAF1 (1% CAF), BaP (12.25 µg/L), CAF0.5 + BaP, and CAF1 + BaP

### Hematological biomarkers

CAF supplementation maintained normal hematological profiles and increased WBC count (Table 6). BaP exposure caused significant (*P* < 0.001) hematological disturbances, including decreased WBCs, RBCs, Hb, and PCV, as well as elevated MCV. CAF addition ameliorated these effects, particularly at the higher dose, reflecting improved physiological resilience. Moreover, these indices were affected by the interaction between CAF supplementation and BaP exposure (*P* < 0.05) except for the count of granulocytes, monocytes, and eosinophils. Furthermore, no significant changes were observed between the acetone and control groups (supplementary Table 3) regarding these biomarkers.

**Table 6:** Hematological biomarkers of *O. niloticus* fed diets supplemented with CAF and/or exposed to BaP for 30 days.

Parameters	WBCs (10^3^/mm^3^)	Granulocytes (10^3^/mm^3^)	Monocytes (10^3^/mm^3^)	Eosinophils (10^3^/mm^3^)	Lymphocytes (10^3^/mm^3^)	Hb (g/dL)	RBCs (10^6^/mm^3^)	PCV (%)	MCHC (g/dL)	MCV (fL)
*Effect of CAF supplementation*										
Non-supplemented	4.13^c^	0.52^b^	0.32^b^	0.20^b^	3.08^b^	6.50^c^	1.73^c^	21.50^c^	29.80	126.89
CAF0.5	3.93^b^	0.53^b^	0.38^ab^	0.24^ab^	3.78^ab^	7.47^b^	1.95^b^	24.00^b^	31.08	123.43
CAF1	5.95^a^	0.66^a^	0.48^a^	0.30^a^	4.21^a^	8.52^a^	2.18^a^	27.17^a^	30.18	124.63
*Effect of BaP exposure*										
Non-exposed	5.73^a^	0.64^a^	0.46^a^	0.31^a^	4.32^a^	8.59^a^	2.26^a^	27.44^a^	31.25	121.65^b^
BaP	4.08^b^	0.50^b^	0.33^b^	0.19^b^	3.06^b^	6.41^b^	1.65^b^	21.00^b^	30.29	128.31^a^
*Interaction effect (CAF supplementation x BaP exposure)*										
Control	5.52^b^	0.60	0.41	0.26	4.25^a^	8.63^a^	2.30^a^	27.53^a^	31.36	119.56^c^
CAF0.5	5.47^b^	0.57	0.42	0.28	4.21^a^	8.16^a^	2.22^a^	26.43^a^	30.89	118.93^c^
CAF1	6.19^a^	0.75	0.56	0.38	4.50^a^	8.93^a^	2.24^a^	28.37^a^	31.49	126.46^c^
BaP	2.73^d^	0.44	0.23	0.16	1.90^c^	4.36^c^	1.15^d^	15.46^c^	28.27	134.21^a^
CAF0.5 + BaP	4.39^c^	0.49	0.35	0.20	3.35^b^	6.76^b^	1.68^c^	21.56^b^	31.37	127.92^b^
CAF1 + BaP	5.11^b^	0.58	0.40	0.22	3.91^b^	8.10^a^	2.11^b^	25.96^a^	31.22	122.79^c^
PSE	0.05	0.02	0.02	0.01	0.13	0.02	0.01	0.22	0.37	1.65
Two-way ANOVA	*P*-values									
CAF supplementation	< 0.001	0.009	0.022	0.034	0.011	< 0.001	< 0.001	< 0.001	0.224	0.430
BaP exposure	< 0.001	0.001	0.006	0.001	< 0.001	< 0.001	< 0.001	< 0.001	0.215	0.009
Interaction	< 0.001	0.526	0.463	0.401	0.033	< 0.001	< 0.001	< 0.001	0.156	0.012

Data (mean) represent tank-level replication (*n* = 4 tanks per treatment group, with 3 fish subsampled per tank to calculate individual tank means). Values carrying various superscripts within the same column are significantly different (two-way ANOVA, Tukey’s HSD test, *P* < 0.05). Overall pooled standard error (PSE) and *P*-values for main effects (CAF supplementation, BaP exposure) and their interaction are presented.

Groups: control, CAF0.5 (0.5% CAF), CAF1 (1% CAF), BaP (12.25 µg/L), CAF0.5 + BaP, and CAF1 + BaP.

### Gene expression

**Fig. 1:**
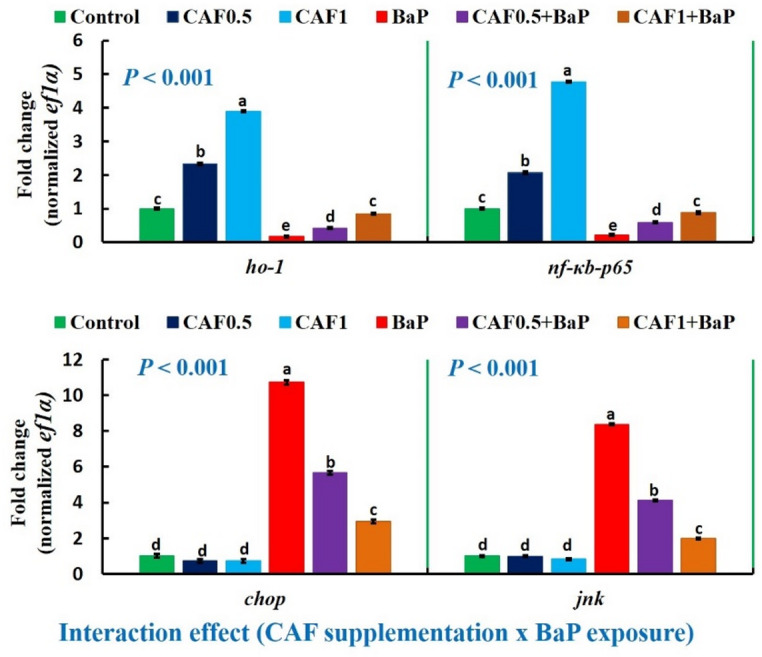
Interaction effect on the expression of splenic *ho-1*, *nf-κb-p65*, *chop*, and *jnk* of *O. niloticus* fed diets supplemented with CAF and/or exposed to BaP for 30 days. Data (mean ± PSE) represent tank-level replication (*n* = 4 tanks per treatment group, with 3 fish subsampled per tank to calculate individual tank means). Bars carrying various superscripts are significantly different based on the interaction effect (two-way ANOVA, Tukey’s HSD test, *P* < 0.05). PSE: pooled standard error. Groups: control, CAF0.5 (0.5% CAF), CAF1 (1% CAF), BaP (12.25 µg/L), CAF0.5 + BaP, and CAF1 + BaP.

CAF upregulated antioxidant and immune-related genes (*ho-1* and *nf-κb-p65*), whereas BaP suppressed these genes and induced pro-apoptotic genes (*chop* and *jnk*) as demonstrated in Fig. 1. CAF co-treatment restored protective gene expression and attenuated pro-apoptotic signaling, indicating molecular-level mitigation of BaP toxicity. Furthermore, the interaction revealed a considerable effect (*P* < 0.001) on the expression of these genes. On the contrary, acetone did not change their expression levels relative to the control group (supplementary Table 4).

### Neuro-stress biomarkers and BaP residues

**Fig. 2:**
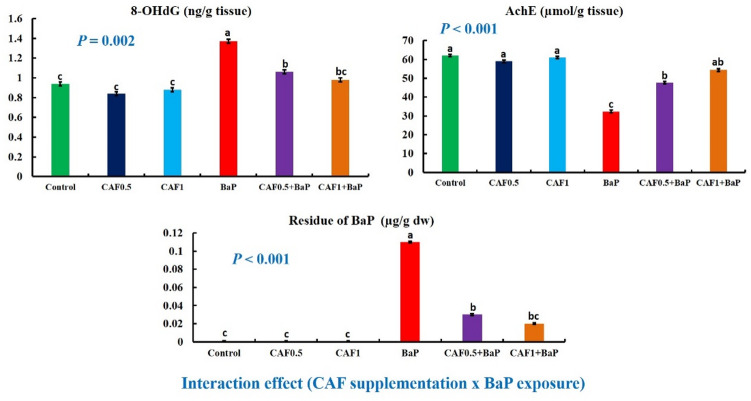
Interaction effect on stress-neuro biomarkers and BaP residues in the muscles of *O. niloticus* fed diets supplemented with CAF and/or exposed to BaP for 30 days. Data (mean ± PSE) represent tank-level replication (*n* = 4 tanks per treatment group, with 3 fish subsampled per tank to calculate individual tank means). Bars carrying various superscripts are significantly different based on the interaction effect (two-way ANOVA, Tukey’s HSD test, *P* < 0.05). PSE: pooled standard error. Groups: control, CAF0.5 (0.5% CAF), CAF1 (1% CAF), BaP (12.25 µg/L), CAF0.5 + BaP, and CAF1 + BaP.

BaP exposure elevated 8-OHdG, accumulated residues, and decreased AchE activity (*P* < 0.001, Fig. 2). CAF supplementation significantly (*P* < 0.001) reduced 8-OHdG and residues, while restoring AchE activity, supporting neuroprotective and detoxifying effects. Additionally, the 8-OHdG and AchE were affected by the interaction (*P* < 0.05) between CAF inclusion and BaP subjection. Addition of acetone did not alter these parameters (supplementary Table 4).

### Histopathological outcomes

**Fig. 3:**
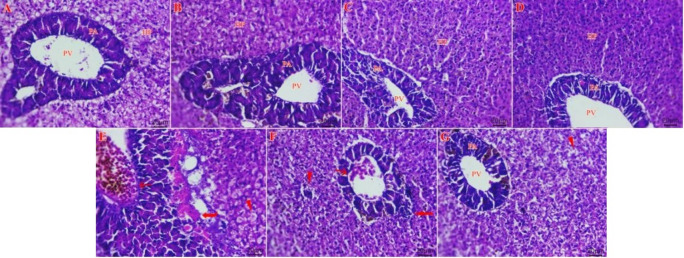
Photomicrograph of liver sections of *O. niloticus* (H&E, 40 ×) fed diets supplemented with CAF and/or exposed to BaP for 30 days. **A**–**D** Control, acetone, CAF0.5, and CAF1 groups show typical structures of hepatocytes, pancreatic acini, and vascular tissues. **E** BaP group shows hydropic degeneration, oncotic necrosis at numerous hepatocytes (red arrowhead), necrotic pancreatic acini (curved arrow), and congested portal vein (arrow). **F** CAF0.5 + BaP group shows apparently normal hepatic and pancreatic parenchyma with a few necrotic numbers of hepatic cells (arrowhead), peripancreatic inflammatory cell aggregates (thick arrow), and a mildly congested portal vein (thin arrow). **G** CAF1 + BaP group shows apparently normal hepatic and pancreatic parenchyma with a few numbers of hydropic degenerated hepatocytes (arrowhead). PA: pancreatic acini. HP: hepatocytes. PV: portal vein. Groups: control, acetone (12.25 µL/L), CAF0.5 (0.5% CAF), CAF1 (1% CAF), BaP (12.25 µg/L), CAF0.5 + BaP, and CAF1 + BaP. Scale Bar: 20 μm.

Histological investigation of the liver showed normal vascular, pancreatic, and hepatic structures in the control, acetone, and CAF-only groups (Fig. 3A–D). BaP exposure caused marked liver damage, including hydropic degeneration and necrosis, pancreatic acinar degeneration, and portal vein congestion (Fig. 3E). Supplementation with CAF improved these alterations in a dose-dependent manner (Fig. 3F and G), with CAF1 + BaP restoring liver and pancreatic architecture almost to normal, supporting its hepato-pancreatic protective potentials.

**Fig. 4:**
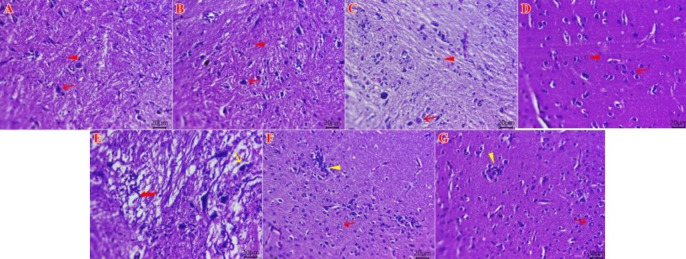
Photomicrograph of brain sections of *O. niloticus* (H&E, 40 ×) fed diets supplemented with CAF and/or exposed to BaP for 30 days. **A**–**D** Control, acetone, CAF0.5, and CAF1 groups show regular architectures of neuronal cell bodies (red thin arrows), glial cells (arrowheads), and neuropil. **E** BaP group shows neuropil vacuolations (curved arrow) and numerous pyknotic neurons with dark, eosinophilic cytoplasm and pyknotic nuclei (yellow thin arrow). **F** and **G** CAF0.5 + BaP and CAF1 + BaP groups show apparently normal neurons (red arrows), glial cells, and vascular tissues, with glial cell aggregation around some degenerating neurons (yellow arrowheads). Groups: control, acetone (12.25 µL/L), CAF0.5 (0.5% CAF), CAF1 (1% CAF), BaP (12.25 µg/L), CAF0.5 + BaP, and CAF1 + BaP. Scale Bar: 20 μm.

Brain histology was normal in the control, acetone, and CAF-only groups (Fig. 4A–D). BaP exposure induced neuropil vacuolation and neuronal degeneration (Fig. 4E), while CAF supplementation markedly preserved neuronal and glial structures and reduced degenerative changes (Fig. 4F and G), indicating its neuroprotective effects.

**Fig. 5:**
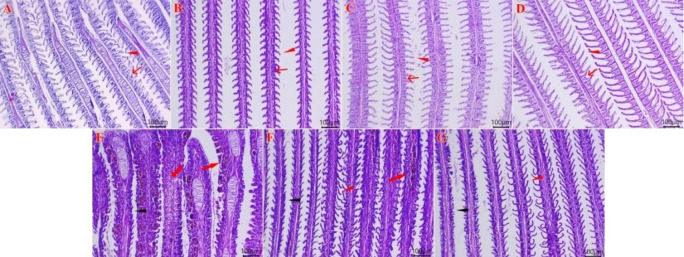
Photomicrograph of gill sections of *O. niloticus* (H&E, 10 ×) fed diets supplemented with CAF and/or exposed to BaP for 30 days. **A**–**D** Control, acetone, CAF0.5, and CAF1 groups show a regular morphological arrangement of primary filaments (red arrows) and secondary filaments (red arrowheads), as well as normal vascular tissue. **E** BaP group shows a denuded surface of most primary filaments (black arrowhead), thickened gill filaments with inflammatory cell infiltrates, congested capillaries (thick arrow), and extravasated erythrocytes (curved arrow). **F** CAF0.5 + BaP group shows apparent regular most gill filaments (red arrowhead) with some stunted secondary filaments (black arrowhead) beside a congested gill capillary (thick arrow). **G** CAF1 + BaP group shows apparent normality, with most gill filaments (red arrowhead) having eroded surfaces, and some primary filaments arising from secondary filaments (black arrowhead). Groups: control, acetone (12.25 µL/L), CAF0.5 (0.5% CAF), CAF1 (1% CAF), BaP (12.25 µg/L), CAF0.5 + BaP, and CAF1 + BaP. Scale Bar: 100 μm.

Gill histology was normal in the control, acetone, and CAF-only groups (Fig. 5A–D). BaP exposure caused severe structural damage, including denuded primary filaments, thickening, inflammatory infiltration, and vascular congestion (Fig. 5E). The observed alterations were reduced by CAF in a dose-related response (Fig. 5F and G). In particular, fish in the CAF1 + BaP group exhibited near-normal gill structure, supporting the protective role of CAF against BaP-induced gill injury.

**Fig. 6:**
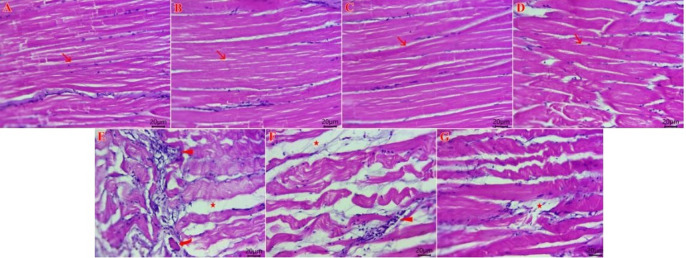
Photomicrograph of skeletal muscle sections of *O. niloticus* (H&E, 40 ×) fed diets supplemented with CAF and/or exposed to BaP for 30 days. **A**–**D** Control, acetone, CAF0.5, and CAF1 groups show normal morphological architectures of multinucleated striated muscle fibers (thin arrows). **E** BaP group shows hyaline degeneration (curved arrow), thickening of interstitial tissues by fibrous connective tissue proliferation (arrowhead), and edema within endomysium (star). **F** CAF0.5 + BaP group shows apparently normal striated muscle fibers with mild strands of fibrous connective tissues with few interstitial inflammatory cell infiltrates (arrowhead) and interstitial edema (star). **G** CAF1 + BaP group shows apparently normal myofibers with mild interstitial edema (star). Groups: control, acetone (12.25 µL/L), CAF0.5 (0.5% CAF), CAF1 (1% CAF), BaP (12.25 µg/L), CAF0.5 + BaP, and CAF1 + BaP. Scale Bar: 20 μm.

In the control, acetone, and CAF-only groups, skeletal muscle displayed normal morphology (Fig. 6A–D). BaP-exposed fish, however, exhibited myofiber degeneration, interstitial edema, and increased connective tissue proliferation (Fig. 6E). CAF supplementation alleviated these changes (Fig. 6F and G), with CAF1 + BaP largely restoring muscle architecture, suggesting that CAF protects against BaP-induced muscular damage.

### Bacterial challenge resistance

**Fig. 7:**
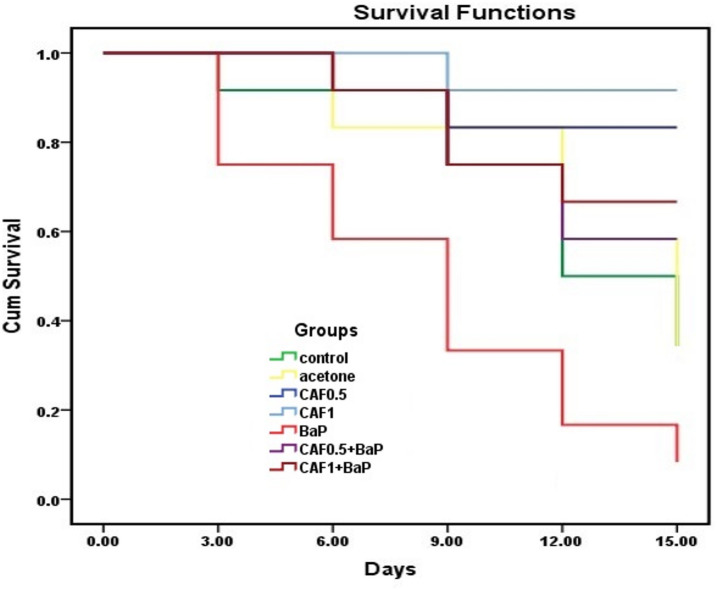
Kaplan–Meier survival curves of *O. niloticus* fed diets supplemented with CAF and/or exposed to BaP for 30 days and followed by infection with *A. hydrophila* for 15 days. Groups: control, acetone (12.25 µL/L), CAF0.5 (0.5% CAF), CAF1 (1% CAF), BaP (12.25 µg/L), CAF0.5 + BaP, and CAF1 + BaP. Survival profiles were statistically analyzed and compared using the log-rank (Mantel–Cox) test (*P* = 0.00002). Data represent cumulative survival across *n* = 4 replicate tanks per group (initially stocked with 3 fish per tank for the challenge phase, totaling 12 fish per treatment group).

BaP-exposed fish showed pronounced signs of *A. hydrophila* infection, such as sluggish movement, scale loss, and skin hemorrhages after 30 days. In contrast, fish in the CAF-supplemented groups exhibited notably milder symptoms, suggesting its potential protective effects. Kaplan–Meier analysis (Fig. 7) revealed that CAF supplementation enhanced survival rates, with CAF1 providing the highest protection, both alone (91.67%) or in the BaP-exposed fish (66.67%), validating enhanced immunological protection.

## Discussion

This study is essential due to the severe toxic effects of BaP on the overall health of fish. Given the limited evidence on nutritional mitigation strategies, this study aimed to evaluate the potential effects of dietary CAF in reducing BaP toxicity in *O. niloticus*. The effects of plants are mainly attributed to their composition of phytochemicals, or secondary metabolites, that provide antioxidant, anti-inflammatory, and antimicrobial properties^68,69^. These phytochemicals include glucosinolates, carotenoids, polyphenols, alkaloids, terpenes, and vitamins^73,74^. The amount of each phytochemical in a plant depends on the environment in which it was grown, the method of cultivation, and the method used to process it^70–72^. In the present study, GC–MS profiling revealed that the CAF extract was highly rich in various isothiocyanates, predominantly phenethyl isothiocyanate (19.20%), 3-butenyl isothiocyanate (15.90%), 4-pentenyl isothiocyanate (13.50%), and benzyl isothiocyanate (11.20%), alongside minor amounts of fatty acids and terpenes. Among these compounds, isothiocyanates are characteristic functional secondary metabolites of cruciferous vegetables^75,76^, formed via enzymatic hydrolysis of glucosinolates^77^. Isothiocyanates possess profound biological importance; their electrophilic character is widely reported to allow them to readily covalently bind to cysteine residues in proteins, potentially activating nuclear factor erythroid 2–related factor 2 (*NRF2* protein). This activation is hypothesized to enhance antioxidant defense, regulate inflammatory responses, and upregulate detoxification-related genes^76,78–81^. Therefore, the high relative abundance of total isothiocyanates identified in CAF may explain the reduced mortality and alleviated BaP toxicity, which is likely associated with a speculative greater activation of the host’s detoxification system and immune response.

Biochemical markers such as hepatic enzymes (ALT, ALP, and AST), renal indices (creatinine and urea), and stress indicators (glucose and cortisol) are widely used to assess toxicological impacts in fish^82,83^. These markers reflect early organ dysfunction and physiological stress^84^. In the present study, all of the evaluated biomarkers were significantly elevated in the BaP-exposed group; however, protein fractions were significantly decreased. These findings are consistent with previous studies reporting stress responses in BaP-exposed fish^85^. The primary cause of BaP toxicity is widely associated with the formation of reactive metabolites after bioactivation of BaP by cytochrome P450 enzymes^86^. The covalent binding of reactive metabolites to macromolecules and their production of ROS can result in oxidative damage and the dysfunction of cells. Consequently, hepatic enzymes leak into the bloodstream^23,87,88^. Also, renal damage as a result of the oxidative damage to glomerular and tubular structures may lead to increased creatinine and urea levels^89,90^. Elevated cortisol levels further confirm the occurrence of a stress response due to BaP exposure^91^. In contrast, CAF supplementation markedly improved these biochemical alterations. This response is possibly attributed to the rich profile of isothiocyanates in CAF, which may increase antioxidant activity and minimize lipid peroxidation^35,92,93^. Additionally, these volatile constituents might support detoxification pathways, promoting the biotransformation and elimination of toxic substances^35,94^. There have been similar protective effects reported from other Brassica plant extracts that have been shown to attenuate damage caused by some toxicants (such as arsenic) to the liver and kidneys of rats^95^. Even though there are not many investigations about the impact of Brassica supplements in fish, some existing studies have indicated possible detoxification advantages. Nevertheless, more research is required to further substantiate the efficiency of these supplements under the influence of BaP.

Oxidative stress and immune dysfunction are key biomarkers of pollutant toxicity, commonly evaluated via markers such as MDA, CAT, SOD, and GST^96–98^ and immune parameters including lysozyme, NO, and IgM^99,100^. The current study documented the immunotoxic effects of BaP, which led to reduced antioxidant and immune defenses alongside elevated MDA levels. Antioxidant capacity was restored by CAF supplementation, and immune parameters were observed to be improved, especially when using higher levels of supplementation. The overproduction of ROS from BaP exposure negatively affects redox balance in the body and may result in the degradation of cellular structures. While the antioxidant enzymes are expressed in response to the production of ROS, there is a limit as to how much they can be produced before there is complete depletion^19,89,101,102^. This oxidative imbalance might impair immune function by potentially inhibiting the *NF-κB* signaling, which is crucial for innate immunity. As a result, there is a decrease in NO production and globulin responses, along with a reduction in lysozyme activity^21,87^. To date, there is very limited evidence that supports the activity of CAF in fish; however, mechanistic evidence from other species supports the protective effects of Brassica vegetables. These impacts are thought to be primarily correlated with the abundance of bioactive compounds, particularly the glucosinolate-derived isothiocyanates detected in our study^103–106^. These constituents are thought to activate the *NRF2* pathway through an interaction with Kelch-like ECH-associated protein 1 (*KEAP1*), leading to a possible increase in the expression of antioxidant-related genes^107,108^, as previously suggested in models using purified cruciferous bioactive compounds^109,110^. Additionally, these compounds appear to be involved in regulating *NF-κB* and other inflammatory signaling pathways^111,112^. Collectively, these mechanisms may enhance antioxidant defense and immune responses (lysozyme, NO, and IgM)^113–115^.

Hematological parameters are reliable indicators of toxicological stress in fish and how the overall and physiological system is responding to toxic substances^116^. In this study, BaP exposure significantly impaired hematological indices, whereas CAF supplementation restored these parameters, particularly in the CAF1 + BaP group. BaP may mediate its hematologic toxic effects through the stimulation of aryl hydrocarbon receptors^117^, where this receptor is suggested to be capable of overproducing ROS, which could contribute to oxidative cellular damage as well as hemolytic damage^118–120^. The oxidative stress that occurs as a result of the action of BaP can damage the hematopoietic organs (i.e., kidneys and spleens) and promote apoptosis in these organs^85,102,121^, thereby potentially impairing hematopoiesis and disrupting blood cell homeostasis. The improvement in hematological parameters in groups fed diets supplemented with CAF may be attributed to its high concentration of bioactive molecules (especially major components like phenethyl and benzyl isothiocyanates), which are documented to support cellular antioxidant defenses^73,92,103–106^. Similar protective effects of cruciferous diets have been reported against other pollutants^34,35^. In the CAF1 + BaP treatment, the findings of increased Hb, RBCs, and PCV and the decline in MCV appear to indicate the development of a compensatory microcytic erythrocyte response during the recovery phase from BaP-induced hemolysis^116^. In this context, such a microcytic response might develop as a result of rapid erythropoiesis (as a result of residual oxidative stress), creating smaller-sized but high-density Hb-containing erythrocytes, possibly due to the general promotion of antioxidant and detoxification enzyme synthesis associated with CAF components^122,123^. MCHC remained unchanged, indicating a relatively stable Hb synthesis despite variations in cell size and number^124^.

Gene expression markers used to measure oxidative stress, inflammation, and apoptosis after exposure to pollutants in fish include *ho-1*, *nf-κb-p65*, *jnk*, and *chop*. *ho-1* is a cytoprotective factor via *NRF2* pathway activation associated with the enhancement of antioxidant defense mechanisms and reduction of ROS accumulation^125–127^. *nf-κb-p65* is a principal regulator of the inflammatory response that is activated by oxidative stress^128,129^. *jnk* and *chop* are crucial mediators of stress signalling; *jnk* is part of the oxidative stress response, while *chop* is used as a marker of endoplasmic reticulum (ER) stress and apoptosis^130–134^. In the present study, BaP exposure caused a downregulation of *ho-1* and *nf-κb-p65*, as well as an upregulation of *jnk* and *chop*; this shifting of response from adaptive antioxidative response to ER stress/apoptotic signaling recommends a potential loss of both antioxidative and inflammatory signaling pathways associated with prolonged oxidative stress, which may limit the ability for cells to adapt^135^. Similar findings have also been reported regarding impaired detoxification and stress-response pathways caused by chronic BaP exposure to fish models^136,137^. Activation of JNK and CHOP proteins, leading to apoptosis, has been widely associated with sustained oxidative stress and ER stress^132,133^. In contrast, supplementing with CAF counteracted these alterations, bringing gene expression levels closer to baseline values, which was most pronounced in the CAF1 + BaP group. This effect is potentially mediated by the identified isothiocyanates, which are known from literature to induce antioxidant and detoxification systems and influence stress responses. This likely contributed to the observed upregulation of *ho-1* and probable modification to the *jnk* and *chop* pathways (inflammatory and ER stress)^138^. These findings further support the hypothesized role of CAF as a regulator of oxidative stress and detoxification pathways under the specified experimental conditions^34,93^.

Altered molecular structures are evident from downstream biomarkers of cell injury, such as 8-OHdG and AchE activity^139,140^. In this study, BaP exposure increased 8-OHdG and inhibited AchE, suggesting potential oxidative DNA damage and neurotoxicity, which aligns with previous studies^141,142^. The use of CAF reduced levels of 8-OHdG and restored AchE activity, indicating a reduction of oxidative DNA injury and neurotoxicity. This protective impact may be mediated through the identified isothiocyanates, which can modulate Phase II detoxifying enzymes, potentially contributing to a decreased production of ROS and subsequent decreased oxidative injury^73,92,104,105^. They may also influence the Phase I/II balance of enzymes, which could help lower the bioactivation of BaP and enhance excretion of BaP metabolites^32,74,76^. However, direct evidence of CAF reducing BaP bioaccumulation remains limited and warrants further investigation.

The molecular and biochemical changes caused by BaP finally lead to destructive alterations in tissues that can be assessed histopathologically^143^. In the current study, BaP caused significant histopathological changes to multiple fish tissues, including the liver, brain, gill, and muscles. The lipophilic nature of BaP and its high bioaccumulation capacity potentially promote its retention in metabolically active tissues, particularly those of metabolically active tissues, including the liver and kidneys, which is associated with marked histopathological changes and the induction of degenerative and inflammatory lesions^144^. This observation was also supported by high bioaccumulation levels of BaP in fish muscles, suggesting possible public health issues. These findings are in agreement with previous reports^23,145,146^. CAF supplementation significantly alleviated these histopathological alterations, supporting the maintenance of tissue and cellular structure. The mechanism behind this effect may be mediated by the bioactive compounds identified in CAF, such as major isothiocyanates, which are known to enhance antioxidant defenses and support hepatic detoxification mechanisms^73,92,103,147^. Although studies directly addressing the impacts of CAF against BaP-induced histopathological damage or its role in reducing BaP bioaccumulation in fish are scarce, further research is warranted to better elucidate this protective potential. Nevertheless, considering the small number of samples used in assessing the tissue changes (*n* = 3 fish per group, sampled randomly from the experimental replicates to evaluate qualitative changes), the histopathological results are to be taken with caution. The tissue changes are shown here as qualitative evidence to support that BaP-induced toxicity was followed by cell protection due to CAF.

Given that BaP exposure causes oxidative stress, immunological toxicity, and tissue damage, it may increase fish susceptibility to secondary infections^148^. This trend was strongly suggested in the current study, where the mortality rate in BaP-exposed fish infected with *A. hydrophila* reached 91.67%. This high mortality is likely associated with the combined functional, histological, immunological, and oxidative impairments observed in the BaP-exposed group. The study by Carlson et al.^149^ demonstrated that BaP-induced suppression in Japanese medaka (*Oryzias latipes*) affects their ability to resist *Yersinia ruckeri* infection. In contrast, CAF supplementation to the diet of fish exposed to BaP reduced mortality rates to 33.33% in the CAF1 + BaP group and 41.67% in the CAF0.5 + BaP group, pointing toward a probable improved resistance to *A. hydrophila*.

This study demonstrated the potential effects of CAF in mitigating BaP levels in *O. niloticus*; however, several limitations should be acknowledged. The high BaP concentration used represents an extreme exposure scenario rather than typical environmental conditions. Thus, it may not represent moderate or chronic exposure levels normally encountered in aquaculture systems. Consequently, these findings may have limited applicability to long-term, low-dose exposure scenarios, where sublethal effects are more relevant. However, the protective effects observed under conditions of severe exposure to CAF emphasize its potential applications in heavily polluted surroundings.

In addition, this work is a lab-based study that, although controlled, does not fully replicate all aspects of natural aquaculture environments. Factors such as fluctuation in water quality, presence of multiple pollutants, temperature variation, and natural microflora communities can influence BaP metabolism and the efficacy of CAF. Another significant limitation of the current research concerns the absence of analysis for BaP concentrations during the whole exposure period. Although a semi-static renewal method has been used for minimizing the variability of concentration, the hydrophobic characteristics of BaP can cause adsorption processes on the walls of tanks, binding to organic material, or even degradation. Thus, real exposure concentrations may vary from nominal values. Accordingly, future investigations are recommended with the analytical monitoring of BaP to strengthen the experimental model reproducibility.

Moreover, a key limitation is that GC–MS provided only a semi-quantitative profile based on peak area percentages via NIST library matching, lacking absolute quantification of specific bioactive groups (e.g., glucosinolates or phenolics) verified by reference standards. Consequently, our mechanistic interpretations regarding specific pathways (e.g., *nrf2*) should be interpreted as potential correlations rather than definitive causative conclusions. The possible interaction between the solvent used in BaP application and CAF was not assessed, and hence, this could be considered a confounding variable. Furthermore, while the CAF was washed to reduce surface contaminants, a specific pesticide residue analysis was not conducted. Although this was beyond the study’s scope, using a consistent source for all groups ensures that the protective effects are primarily due to the plant’s bioactive compound.

Another technical limitation is the lack of species-specific validation for the commercial assay kits; although these kits were applied strictly according to the manufacturer’s guidelines and are widely used in fish toxicology, this necessitates caution in interpreting the results in terms of absolute values. In addition to the lack of a semi-quantitative histopathology score system, this is compounded by the small number of samples used for evaluating tissue damage (*n* = 3 per treatment). These histopathological results can therefore be interpreted only as qualitative support for the observed damage caused by BaP exposure and protection conferred by CAFs.

Finally, while the sample size used in the experiment is consistent with existing standards in fish toxicology (*n* = 4 tanks), increasing the replicates in future research may improve the robustness of the statistics. Given that the study was conducted in a controlled environment, it ensures that any variable is considered to help establish a clear baseline trend; nevertheless, scaling up the models or applying them on the field scale will play a vital role in applying these promising findings in the natural ecosystems. Future research efforts should also evaluate lower, environmentally realistic levels of BaP, as well as the impacts of contaminant mixture.

## Conclusion

In summary, exposure to BaP resulted in major clinical signs, altered biochemistry, blood cell levels, free radical production, decreased immune response, DNA mutations, and a variety of tissue changes (histopathology). Conversely, the dietary CAF at 0.5% and 1% mitigated BaP toxicity by reducing fish mortality rates, restoring the biochemical and hematological parameters, and enhancing fish antioxidant and immune response. In addition, CAF modulated the transcription of stress-associated genes and reduced BaP accumulation in fish tissues, thereby alleviating tissue damage. Most importantly, CAF supplementation increased the resistance of fish to a second infection with *A. hydrophila.* In conclusion, these results confirm the application of dietary CAF as an effective approach for promoting the health, survival, and production capacity of *O. niloticus* in polluted aquaculture conditions. From a practical standpoint, using cauliflower CAF that consists of these bioactive substances in the aquatic feed could prove effective for increasing their resistance to xenobiotics. This will eventually secure food security as well as reduce financial risks involved with pollution-related diseases.

## Supplementary Information

Below is the link to the electronic supplementary material.

Supplementary Material 1

## Data Availability

All data generated or analyzed during this study are included in this article.
