# Correction: Dietary cauliflower (*Brassica oleracea* var. botrytis) mitigates benzo[a]pyrene-induced oxidative stress, immune dysfunction, and tissue damage in Nile tilapia

**DOI:** 10.1038/s41598-026-64915-9

**Published:** 2026-08-05

**Authors:** Zeinab El-Bouhy, Afaf N. Abdel Rahman, Fatma A. S. Mohamed, Mohamed W. A. Elashhab, Tarek Khamis, Rasha M. Reda

**Affiliations:** 1 Department of Aquatic Animal Medicine, Faculty of Veterinary Medicine, Zagazig University, PO Box 44511, Zagazig, Egypt; 2 Department of Aquatic Health and Diseases, National Institute of Oceanography and Fisheries, NIOF, Cairo, Egypt; 3 Department of Pharmacology, Faculty of Veterinary Medicine, Zagazig University, PO Box 44511, Zagazig, Egypt

Correction to: *Scientific Reports* 10.1038/s41598-026-57004-4, published online 17 June 2026

The original version of this Article contained errors.

In the Materials and methods, under the subheading ‘Fish rearing and animal ethics’, the size of the fish tank was incorrect. As a result,

“For 15 days, the fish were housed in well-ventilated 100 L aquaria (80×30×40 cm; 10 fish per tank) containing dechlorinated water.”

now reads:

“For 15 days, the fish were housed in well-ventilated 100 L aquaria (80×60×40 cm; 10 fish per tank) containing dechlorinated water.”

Under the subheading ‘Stress-neuro assays’, the Cat. No. of the My-Biosource commercial kit was incorrect. As a result,

“Furthermore, using a commercial kit (Cat. No. MBS280290) from My-Biosource and a 450 nm wavelength, the activity of AchE was calculated as per a previous technique.”

now reads:

“Furthermore, using a commercial kit (Cat. No. MBS035436) from My-Biosource and a 450 nm wavelength, the activity of AchE was calculated as per a previous technique.”

Furthermore, the Results section under the subheading ‘Oxidant/antioxidant and immune indices’, contained a text error. As a result,

“CAF diets increased antioxidant (CAT, GST, and SOD) and immune (antiprotease, lysozyme, NO, and IgM) activities in a dose-dependent manner, with no effect on MDA level (Table 5).”

now reads:

“CAF diets increased antioxidant (CAT, GST, and SOD) and immune (antiprotease, lysozyme, NO, and IgM) activities in a dose-dependent manner, and decreased MDA level (Table 5).”

Moreover, Table 4 contained an error where the *P-value* in the column ‘AST’ for the Parameter ‘Interaction’ was incorrect.

The incorrect and corrected values appear below:

*Incorrect:*

**Table:** 

Parameters	AST (U/L)
Interaction	0.047

*Correct:*

**Table:** 

Parameters	AST (U/L)
Interaction	0.067

Finally, Supplementary Tables 1 and 2 contained two unit errors. The original Supplementary information file is provided below.

The original Article and accompanying Supplementary Information file have been corrected.

## Supplementary Information

Supplementary Information.

