# Supplementary material for: Dietary cauliflower (Brassica oleracea var. botrytis) mitigates benzo[a]pyrene-induced oxidative stress, immune dysfunction, and tissue damage in Nile tilapia
Source: Sci Rep. 2026 Jun 17;16:18854. doi: 10.1038/s41598-026-57004-4 (PMC13276132; doi:10.1038/s41598-026-57004-4)
Supplement: Supplementary file 1 — Supplementary Material 1 [file 41598_2026_57004_MOESM1_ESM.docx]

| **Supplementary Table 1** Effect of acetone exposure on biochemical biomarkers of *O. niloticus* for 30 days | | | |
| --- | --- | --- | --- |
| Parameters | Control | Acetone | *P* value |
| Creatinine (mg/dL) | 0.75±0.03 | 0.78±0.03 | 0.58 (ns) |
| Urea (mg/dL ) | 12.12±0.21 | 12.86±0.94 | 0.49 (ns) |
| ALT (U/L) | 21.00±1.53 | 19.00±1.15 | 0.35 (ns) |
| ALP (U/L) | 35.00±0.58 | 37.33±1.45 | 0.21 (ns) |
| AST (U/L) | 18.00±0.58 | 19.33±0.88 | 0.27 (ns) |
| Albumin (g/dL) | 2.23±0.07 | 2.35±0.13 | 0.48 (ns) |
| Total protein (g/dL) | 3.25±0.04 | 3.44±0.14 | 0.26 (ns) |
| Globulin (g/dL) | 1.02±0.05 | 1.09±0.02 | 0.25 (ns) |
| Glucose (mg/dL) | 60.00±1.53 | 62.00±2.08 | 0.48 (ns) |
| Cortisol (ng/mL) | 48.00±1.53 | 48.00±0.58 | 1.00 (ns) |
| Data (mean ± SE) represent tank-level replication (*n* = 4 tanks per group, with 3 fish subsampled per tank to calculate individual tank means). Differences between control and acetone groups were analyzed using an independent samples t-test. ns = not significant (*P* ˃ 0.05). Groups: control, acetone (12.25µL/L) | | | |

| **Supplementary Table 2** Effect of acetone exposure on antioxidant-immune responses of *O. niloticus* for 30 days | | | |
| --- | --- | --- | --- |
| Parameters | Control | Acetone | *P* value |
| MDA (nmol/mL) | 12.23±0.21 | 12.55±0.36 | 0.50 (ns) |
| CAT (U/mL) | 158.33±0.88 | 158.33±2.60 | 1.00 (ns) |
| GST (U/mL) | 7.06±0.35 | 7.12±0.03 | 0.87 (ns) |
| SOD (U/mL) | 5.85±0.26 | 5.26±0.15 | 0.13 (ns) |
| Antiprotease activity (U/mL) | 0.51±0.01 | 0.52±0.03 | 0.68 (ns) |
| Lysozyme (U/mL) | 23.66±0.88 | 23.67±1.45 | 1.00 (ns) |
| NO (µmol/L) | 157.66±1.76 | 155.00±3.21 | 0.51 (ns) |
| IgM (mg/dL) | 21.66±1.76 | 22.00±1.52 | 0.89 (ns) |
| Data (mean ± SE) represent tank-level replication (*n* = 4 tanks per group, with 3 fish subsampled per tank to calculate individual tank means). Differences between control and acetone groups were analyzed using an independent samples t-test. ns = not significant (*P* ˃ 0.05). Groups: control, acetone (12.25 µL/L) | | | |

| **Supplementary Table 3** Effect of acetone exposure on hematological biomarkers of *O. niloticus* for 30 days | | | |
| --- | --- | --- | --- |
| Parameters | Control | Acetone | *P* value |
| WBCs (10^3^/mm^3^) | 5.52±0.05 | 5.63±0.03 | 0.13 (ns) |
| Granulocytes (10^3^/mm^3^) | 0.60±0.03 | 0.64±0.01 | 0.26 (ns) |
| Monocytes (10^3^/mm^3^) | 0.41±0.01 | 0.42±0.01 | 0.59 (ns) |
| Eosinophils (10^3^/mm^3^) | 0.26±0.01 | 0.28±0.01 | 0.15 (ns) |
| Lymphocytes (10^3^/mm^3^) | 4.25±0.01 | 4.26±0.03 | 0.68 (ns) |
| Hb (g/dL) | 8.63±0.23 | 8.17±0.18 | 0.19 (ns) |
| RBCs (10^6^/mm^3^) | 2.30±0.01 | 2.28 ± 0.02 | 0.32 (ns) |
| PCV (%) | 27.53±0.79 | 26.30±0.32 | 0.22 (ns) |
| MCHC (g/dL) | 31.36±0.43 | 31.05±0.34 | 0.59 (ns) |
| MCV (fL) | 119.56±3.86 | 115.56±2.49 | 0.43 (ns) |
| Data (mean ± SE) represent tank-level replication (*n* = 4 tanks per group, with 3 fish subsampled per tank to calculate individual tank means). Differences between control and acetone groups were analyzed using an independent samples t-test. ns = not significant (*P* ˃ 0.05). Groups: control, acetone (12.25 µL/L) | | | |

| **Supplementary Table 4** Effect of acetone exposure on the expression of splenic immune genes (fold change normalized *ef-1α*) and stress-neuro biomarkers of *O. niloticus* for 30 days | | | |
| --- | --- | --- | --- |
| Parameters | Control | Acetone | *P* value |
| *ho-1* | 1.00±0.01 | 1.01±0.05 | 0.92 (ns) |
| *nf-κb-p65* | 1.01±0.07 | 0.99±0.01 | 0.82 (ns) |
| *chop* | 1.00±0.02 | 1.04±0.01 | 0.28 (ns) |
| *jnk* | 1.00±0.07 | 1.10±0.04 | 0.29 (ns) |
| 8-OHdG (ng/g tissue) | 0.94±0.02 | 0.91±0.03 | 0.58 (ns) |
| AchE (µmol/g tissue) | 62.05±2.27 | 62.05±1.51 | 1.00 (ns) |
| Data (mean ± SE) represent tank-level replication (*n* = 4 tanks per group, with 3 fish subsampled per tank to calculate individual tank means). Differences between control and acetone groups were analyzed using an independent samples t-test. ns = not significant (*P* ˃ 0.05). Groups: control, acetone (12.25 µL/L) | | | |
